# Development and validation of the 6-dye lyophilized fluorescent multiplex PCR system

**DOI:** 10.1371/journal.pone.0356258

**Published:** 2026-08-27

**Authors:** Yuling Cui, Zhe Zhang, Hao Yu, Ming Ji, Lina Jia, Wen Song, Jing Wang, Lei Shang, Xue Bai

**Affiliations:** 1 Key Laboratory of Forensic Genetics of Ministry of Public Security, Institute of Forensic Science, Ministry of Public Security, Beijing, China; 2 Hebei Key Laboratory of Forensic Medicine, College of Forensic Medicine, Hebei Medical University, Shijiazhuang, China; Texas A&M University, UNITED STATES OF AMERICA

## Abstract

Aiming to address the issues associated with traditional liquid STR reagents—such as dependence on cold-chain storage, cumbersome operation, and unsuitability for on-site rapid detection—this study has successfully developed a six-color fluorescent lyophilized multiplex amplification reagent. The reagent includes 29 autosomal STR loci and 1 gender identification locus. By screening high-tolerance Taq polymerase, optimizing lyoprotectant formulations, and developing a lyophilization process based on thermodynamic analysis, stable ambient-temperature storage of key reagent components has been achieved. Performance validation demonstrates that the lyophilized reagent exhibits high sensitivity (100% detection at 0.125 ng DNA), remains stable for 60 days at room temperature, rehydrates rapidly (<1 second), and shows some tolerance to common inhibitors. Benefiting from the “whole-volume loading” advantage, its detection capability for trace samples surpasses that of traditional liquid reagents. In parallel comparison with similar liquid reagents, it achieves completely consistent locus detection rates (100%), fully meeting the requirements for individual identification. With simple operation and a ready-to-use, disposable design, this reagent effectively overcomes the reliance on cold-chain logistics. It provides robust technical and reagent support for rapid on-site forensic testing, emergency response, grassroots applications, and DNA database construction, demonstrating significant application prospects.

## 1. Introduction

Short Tandem Repeats (STRs), as highly polymorphic genetic markers in the human genome, exhibit significant characteristics for individual identification. DNA from different tissues within the same individual shows complete consistency in STR typing, while the number of core repeats at identical STR loci varies substantially among different individuals, with the probability of two individuals sharing an identical STR profile being on the order of one in trillions. Leveraging this property, forensic scientists can achieve precise individual identification and kinship analysis. This is done by detecting STR profiles from biological evidence left at crime scenes. Owing to its high sensitivity and specificity, STR detection technology dominates the forensic genetics market and remains the gold standard for individual identification and paternity testing.

Currently, numerous commercial kits targeting STRs are available on the market, most of which are liquid-based reagents. However, these reagents present several limitations: First, they require refrigerated transportation and storage, restricting their application in basic-level laboratories and remote areas and incurring high cold-chain costs. Second, as aqueous suspensions, they are unstable and must be prepared immediately before use. Third, cumbersome procedures involving multiple pipetting steps may lead to experimental errors or increased contamination risks. Fourth,for trace samples, the template volume that can be added to the system is limited. Lyophilization technology involves freezing the moisture in a sample into ice crystals at low temperatures, followed by the application of appropriate heat under vacuum conditions to sublimate the ice directly from the solid to gas phase, bypassing the liquid phase, thereby achieving dehydration [1,2]. Applying this technology to PCR liquid reagents to form lyophilized reagents can effectively address the above issues.

The advantages of lyophilized reagents include a pre-packaged, single-tube system enabled by innovative lyophilization processes, allowing storage and transportation at ambient temperature. The single-tube, ready-to-use format supports one-time use and disposable operation, avoiding freeze-thaw cycles. The simple procedure—only requiring rehydration with water followed by the addition of the sample—shortens processing time, reduces contamination risks, and lowers labor costs. Furthermore, lyophilized reagents enable higher template loading, making them suitable for trace samples and improving detection rates. Their portability also makes them ideal for extreme environments such as disaster zones and battlefields in emergency and disaster response scenarios [3–5]. Based on lyophilization technology, this study successfully developed a lyophilized 6-dye multiplex amplification reagent targeting 30 autosomal loci, expanding the application scenarios of STR amplification reagents.

## 2 Materials and methods

### 2.1 Samples

Sample 9947A was purchased from Nuhigh Biotechnologies Co. Ltd. Species-specific samples were obtained from previously collected specimens in the laboratory, while routine casework materials included blood cards. The ethical review application for this study was approved by the Scientific Research Ethics Committee of the Institute of Forensic Science under the Ministry of Public Security (Approval No.: 2023−038).

### 2.2 Methods

#### 2.2.1 Loci selection and primer optimization.

The lyophilized reagent developed in this study was based on the DNATyper™ 30 liquid reagent, with subsequent primer optimization for specific loci to meet the stability requirements of the lyophilization process. Regarding the primers, preliminary lyophilization tests were conducted. While the original primers for some loci from DNATyper™ 30 were retained, those for certain loci—specifically D6S477, vWA, FGA, D19S433, Penta E, D10S1435, and D8S1179—exhibited reduced amplification efficiency after lyophilization and therefore required redesign. For the loci requiring primer sequence modifications, we employed software such as Primer Premier 5 (Premier Biosoft, California, United States) and Oligo 7 (Molecular Biology Insights, Colorado, United States) for primer design.

#### 2.2.2 PCR mix development.

This study introduced modifications to the PCR master mix to optimize it for lyophilization. These improvements included removing glycerol from the mix, adjusting component types and concentrations within the PCR system, selecting a Taq DNA polymerase suitable for lyophilization, and adding it at the optimal concentration for the formulation.

#### 2.2.3 PCR condition development.

Systematic optimization was performed on the PCR amplification protocol of DNATyper™ 30. For the annealing temperature, a gradient with 1°C intervals—specifically 57°C, 58°C, 59°C, 60°C, and 61°C—was tested. The cycling parameters were based on 27 cycles, with incremental adjustments of 1 cycle per gradient.

#### 2.2.4 Lyoprotectant deletion.

Based on literature review [6–8], the lyoprotectants selected for testing included trehalose (Sinopharm Chemical,Shanghai,China), sucrose (Sinopharm Chemical,Shanghai,China), lactose (Sinopharm Chemical,Shanghai,China), mannitol (Sinopharm Chemical,Shanghai,China), polyethylene glycol 8000 (PEG8000; Sangon Biotech,Shanghai,China), polyvinylpyrrolidone (PVP-K30; Sinopharm Chemical,Shanghai,China), glycine (Sinopharm Chemical,Shanghai,China), alanine (Sinopharm Chemical,Shanghai,China), and bovine serum albumin (BSA; Nuhigh Biotech, Jiangsu, China). Prepare and test the impact of different concentrations of a single protectant on amplification efficiency, using 1 ng of 9947A standard DNA as the template. Compare the average peak heights of different loci with specific test concentrations listed in Table 1. Based on the test results, select the appropriate protectant concentration to formulate the final protectant system.

**Table 1:** Lyoprotectants and tested concentration.

Lyoprotectants	Test concentration
**sucrose**	2%, 5%, 8%, 10%, 15%, 20%
**trehalose**	2%, 5%, 8%, 10%, 15%, 20%, 25%
**PVP-K30**	2%, 5%, 10%, 15%, 20%
**mannitol**	2%, 5%, 6%, 7%, 8%, 10%
**PEG8000**	0.25%, 0.3%, 0.35%, 0.4%, 2%, 5%, 8%, 10%
**lactose**	1%, 2%, 5%, 8%, 10%, 15%
**glycine**	0.25%, 0.5%, 1%, 2%, 5%, 8%
**alanine**	0.25%, 0.5%, 1%, 2%, 5%, 8%, 10%

#### 2.2.5 Freeze-drying.

The eutectic temperature (Teu) and glass transition temperature (Tg’) of the system comprising the PCR master mix combined with lyoprotectants were determined by differential scanning calorimetry (DSC). The same sample was measured in triplicate, and the mean was calculated. The Teu and Tg’ were determined to be approximately −22°C and −36°C, respectively (S1 Fig).

**Fig 1:**
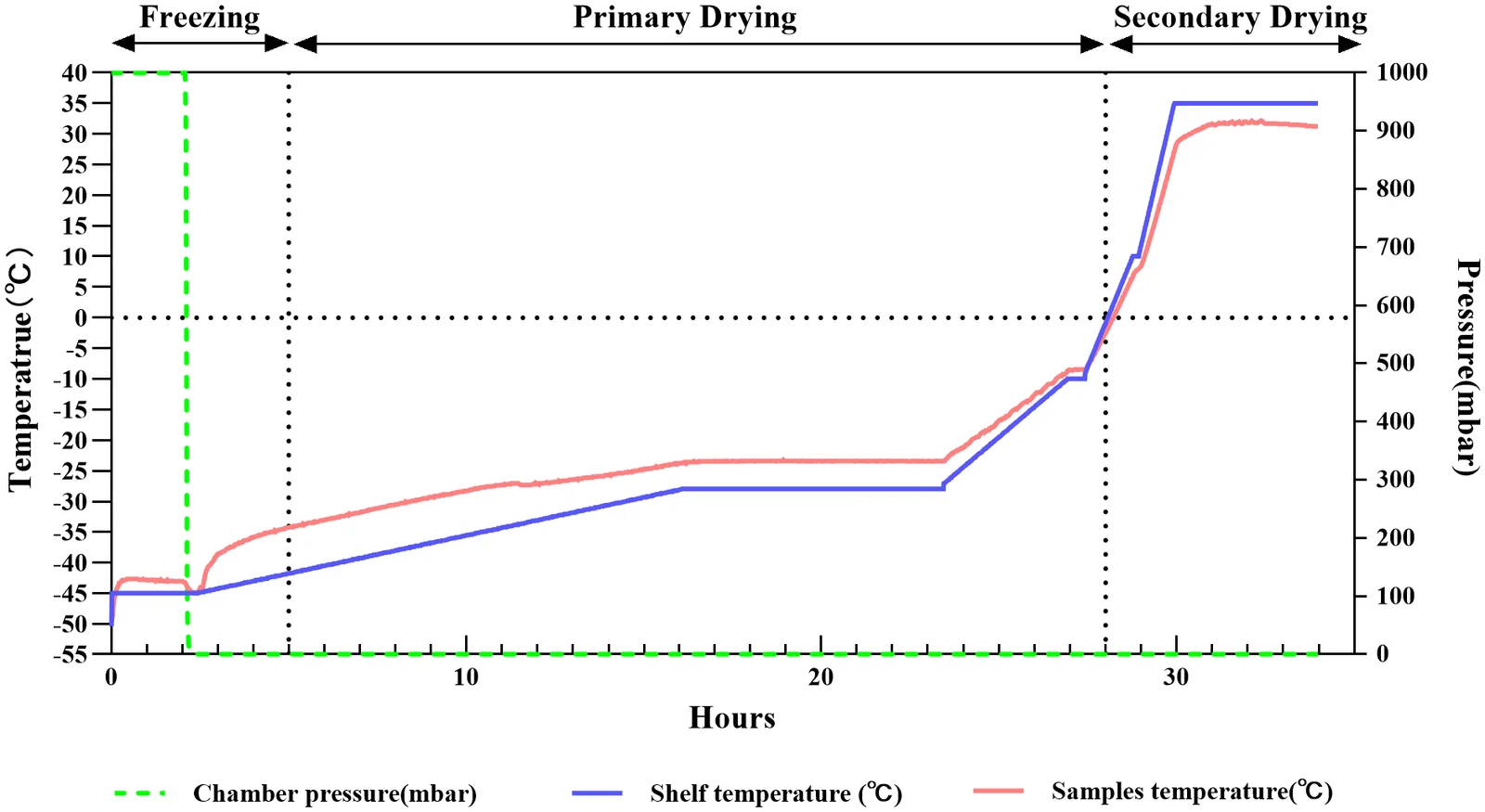
Lyophilization protocol.

Based on these values, the lyophilization protocol was optimized. The pre-freezing temperature was set at −45°C (below both Teu and Tg’) to ensure complete solidification. Primary drying was performed at −28°C with a heating rate of 0.02°Cmin for 440 min, followed by secondary drying at 35°C with a heating rate of 0.16°C/min for 240 min. The chamber pressure was reduced to vacuum during the freezing phase and maintained throughout the entire process. The final protocol was: hold at −45°C for 300 min, ramp to −28°C and hold for 440 min, then ramp to 35°C and hold for 240 min. Detailed results are shown in Fig 1.

### 2.3 Verification of the lyophilized reagent

#### 2.3.1 Sensitivity study.

This study used female standard DNA 9947A as the template, which was serially diluted to yield 1 ng, 0.5 ng, 0.25 ng, 0.125 ng, 0.0625 ng, and 0.03125 ng. Three replicates were performed for each concentration, with the mean peak height and peak detection rate used as evaluation criteria.

#### 2.3.2 Stability study.

The lyophilized reagents were stored at room temperature and 37°C for 7, 15, and 30 days. Using 9947A (1 ng) as the template for amplification, three replicate tests were conducted to compare the locus detection rates and mean peak heights.

#### 2.3.3 Species specificity study.

In a 10 μL amplification system, 1 ng of laboratory-collected samples from 13 species (rabbit, chicken, duck, goat, horse, cattle, cat, mouse, monkey, fish, dog, pig, and *E. coli* DNA) was added, with 9947A DNA used as the positive control. Three replicate experiments were performed.

#### 2.3.4 Mixture study.

9947A and 9948 DNA were mixed in the following ratios: 19:1, 18:2, 16:4, 14:6, 12:8, 10:10, 8:12, 6:14, 2:18, and 1:19, with the total DNA concentration of the mixed samples maintained at 1 ng/μL. A 1 μL aliquot of each mixture was added to a 10 μL lyophilized amplification system. Each ratio was tested three times.

#### 2.3.5 Inhibition study.

Four inhibitors—hematin, indigo, humic acid, and EDTA—were used in tolerance tests. In the 10 μL amplification system, the tested concentrations were as follows: hematin at 0.25, 0.5, and 1 mmol/L; indigo at 3, 4, and 5 mmol/L; humic acid at 60, 80, and 120 ng/μL; and EDTA at 0.25, 0.5, and 1 mmol/L. Amplification was performed using 1 ng of 9947A DNA as the template, with three replicates for each condition.

#### 2.3.6 Trace sample analysis capability.

DNA extracted from minute bloodstain cards collected from actual casework was used as the template. Extraction was performed using magnetic bead-based methods, followed by DNA quantification. Amplification tests were conducted with both 10 μL lyophilized reagents and conventional liquid reagents. The lyophilized reagents employed full-volume loading, whereas the liquid reagents used the maximum recommended sample volume. Three replicate experiments were performed, with locus detection rates and mean peak heights statistically analyzed.

#### 2.3.7 Parallel comparison using liquid reagents.

Using the DNATyper™ 30 liquid reagent as a control, corresponding volumes of components were added to the two reagent formulations according to their respective concentrations (Table 2). Amplification efficiency was evaluated using 1 ng of 9947A DNA as the template, with three replicate experiments conducted to compare the overall performance of the two reagent types before and after lyophilization.

**Table 2:** Component volumes in 10 μL formulations of the two reagent types.

Reagents Components(μL)	2.5 × Buffer	5×Buffer	5×Primer	DNA Sample (9947A)	Water
**DNATyper™30 Liquid Reagent**	4	0	2	1	3
**Lyophilized Reagent**	pre-aliquotted^a^			1	9

^a^The pre-aliquotted solids, upon reconstitution, do not contribute to the final volume.

### 2.4 Amplification

PCR amplification was performed using a ProFlex PCR System (Applied Biosystems, Massachusetts, United States). Taking a 10 μL lyophilized reagent system as an example, 9 μL of deionized water and 1 μL of DNA template (9947A) were added to the lyophilized pellet. The cycling parameters were set as follows: 95°C for 8 min, 28 cycles of 95°C for 10 s, 60°C for 2 min, and 60°C for 30 min.

### 2.5 Electrophoresis detection

The amplification products were detected using a 3500xL Genetic Analyzer (Applied Biosystems). A mixture was prepared by combining 8.91 µL of Hi-Di™ formamide (Thermo Fisher Scientific, Massachusetts, United States) and 0.09 µL of the SIZE-500 molecular size standard (Molecular Cloning Laboratories, San Francisco, United States). Subsequently, 1 µL of the PCR product was added to the corresponding well of the capillary electrophoresis plate for analysis.

### 2.6 Statistical analysis

The electrophoretic data were analyzed using the GeneMapper® ID-X software v1.5 (Applied Biosystems). And the analysis threshold was set at 100 RFU. Peak height data were entered and organized using the Excel component of WPS Office. Statistical analyses were performed using GraphPad Prism 10.4 (GraphPad Software, Massachusetts, United States). Data are presented as mean ± standard deviation (SD) from three independent replicates. Normality was assessed using the Shapiro-Wilk test, and homogeneity of variance was verified by Levene's test. Comparisons among multiple groups were performed using one-way analysis of variance (ANOVA) followed by Dunnett's post hoc test, with the 0.125 ng group serving as the control group for sensitivity analysis. For stability and parallel comparison experiments, comparisons between two groups were performed using paired or unpaired Student's t-test as appropriate. All statistical tests were two-sided, and exact p-values were reported. The 95% confidence intervals (CI) for mean differences were calculated and reported alongside the p-values. A p-value < 0.05 was considered statistically significant.

## 3. Results

### 3.1 Amplification system development

The DNATyper™ 30 liquid reagent kit encompasses 29 autosomal loci and one sex-determination locus (Amelogenin), specifically: D5S818, D21S11, D7S820, CSF1PO, D2S1338, D2S441, D3S1358, vWA, D8S1179, D16S539, Penta E, TPOX, TH01, D19S433, D18S51, FGA, D10S1248, D6S1043, D13S317, D12S391, D1S1656, Penta D, D8S1132, D15S659, D3S3045, D19S253, D6S477, D10S1435, and D22S1045, with ILS-500 as the internal size standard (S2 Fig).

To enhance the stability of the liquid DNATyper™ 30 reagent after lyophilization, this study optimized the master mix components (buffer, enzyme, primer, lyoprotectant). Glycerol in the amplification system primarily originates from DNA polymerase, where it protects enzymatic activity. However, since glycerol compromises lyophilization stability [7],it was removed. Based on references [8–11] and existing protocols, six glycerol-free Taq DNA polymerases were evaluated (Table 3). Enzymatic activity before and after lyophilization was assessed via a fluorescent dye method (S3 Fig). Considering activity loss, cake appearance, and amplification efficiency (locus detection rate and peak height), Enzyme No. 6 was selected (Table 4).

**Table 3:** Characteristics of lyophilization-suitable enzymes selected and validated.

No.	Optimal pH	Extension temperature (℃)	Concentration(U/μL)	Amplification rate(s/kb)
**1**	8.5	72	5	60
**2**	8.5	72	5	60
**3**	8.5	72-74	8	60
**4**	--	60	20	15
**5**	--	--	50	--
**6**	7.8-8.3	72	10	60

**Table 4:** Comparison of enzymatic activity for the six DNA polymerases before and after lyophilization.

No.	Enzyme activity		Loss rate
	Actual enzyme concentration(U/μL)	Enzyme concentration after lyophilization(U/μL)	
**1**	25	14.45	42.2%
**2**	10	7.31	26.9%
**3**	15	10.77	28.2%
**4**	17.16	16.9	1.5%
**5**	19.57	14.5	25.9%
**6**	4.57	4.55	1.1%

Enzyme dosage was then optimized through a gradient test with 0.2 U intervals. In the 10 μL amplification system, enzyme amounts of 0.4 U, 0.6 U, 0.8 U, 1 U, and 1.2 U were tested. Based on the experimental results, it was determined that an enzyme concentration of 1 U/10 μL yielded optimal peak heights across all loci. The 1U dosage demonstrated optimal locus detection rates and mean peak heights, establishing it as the final concentration (S4 Fig).

The concentrations of each component in the final amplification buffer are shown in Table 5.

**Table 5:** Components and concentrations of the final PCR amplification buffer.

Component	Concentration
Tris-HCl	375 mmol/L
(NH4)_2_SO_4_	100 mmol/L
Tween-20	0.5 wt%
MgCl_2_	10 mmol/L
BSA	0.5 mg/mL
KCl	15 mmol/L
dNTPs	5 mmol/L
EDTA	0.05 mmol/L
DTT	1 mmol/L

**Fig 2:**
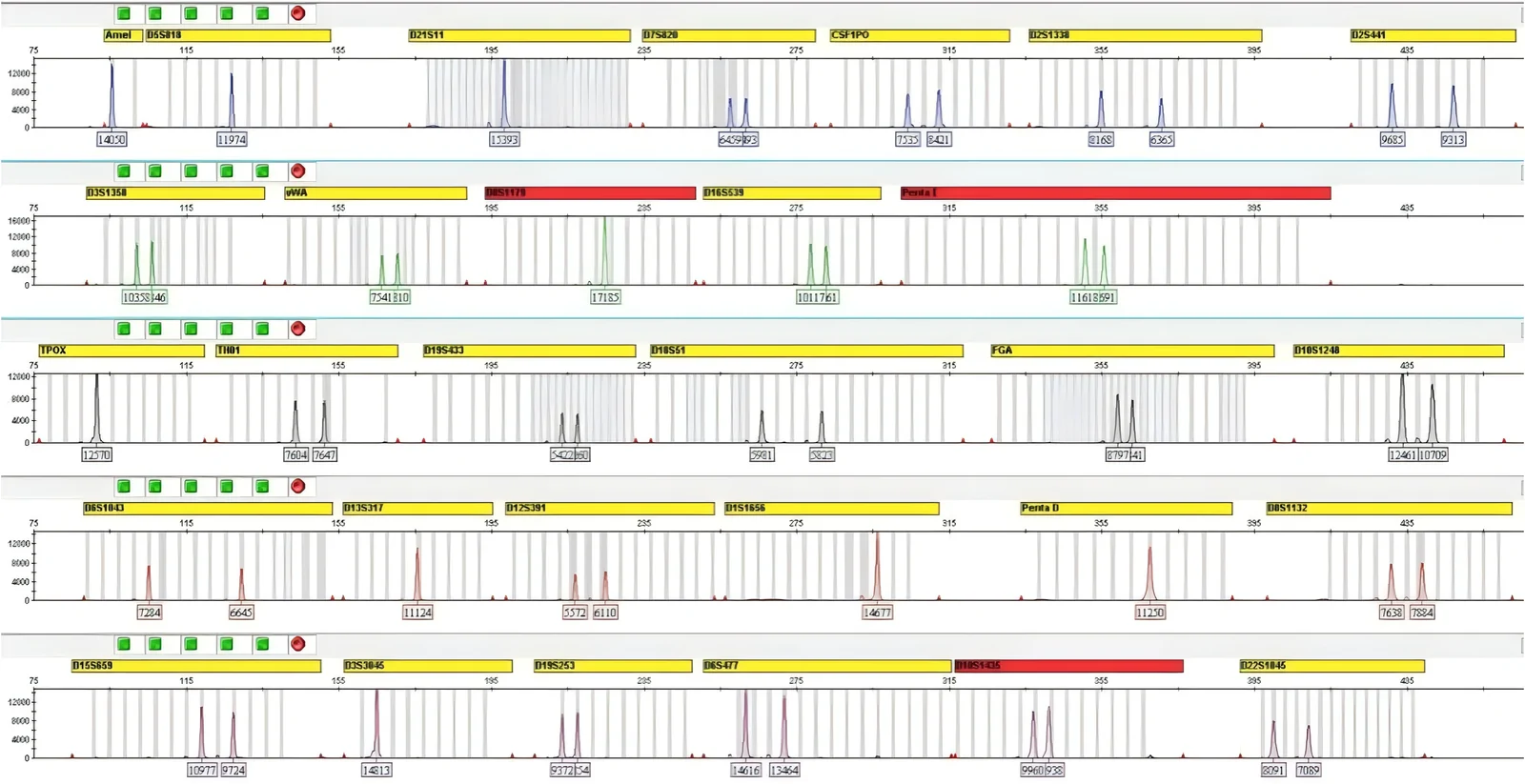
Electropherograms of the redesigned STR loci. Amplification results of 1ng 9947A DNA.

To adapt the lyophilized PCR system, the original primers from the DNATyper^TM^ 30 system were first tested. Some primers (D6S477, vWA, FGA, D19S433, Penta E, D10S1435, and D8S1179) exhibited low peak signals due to their low annealing temperatures. Accordingly, these primers were redesigned. After incorporating the redesigned primers into the overall system, the peak height uniformity was improved. See Fig 2.

Annealing temperature optimization tested gradients from 57°C to 61°C. Evaluation of mean peak heights and detection rates across loci indicated that 60°C was the optimal annealing temperature (S5 Fig). The amplification efficiency was tested over 27–31 cycles (S6 Fig). The figure shows that when the cycle number exceeded 28, nonspecific amplification and double peaks occurred, and the amplification time gradually increased. After a comprehensive evaluation, 28 cycles were selected as the optimal number of amplification cycles.

### 3.2 Freeze-drying system development

**Fig 3:**
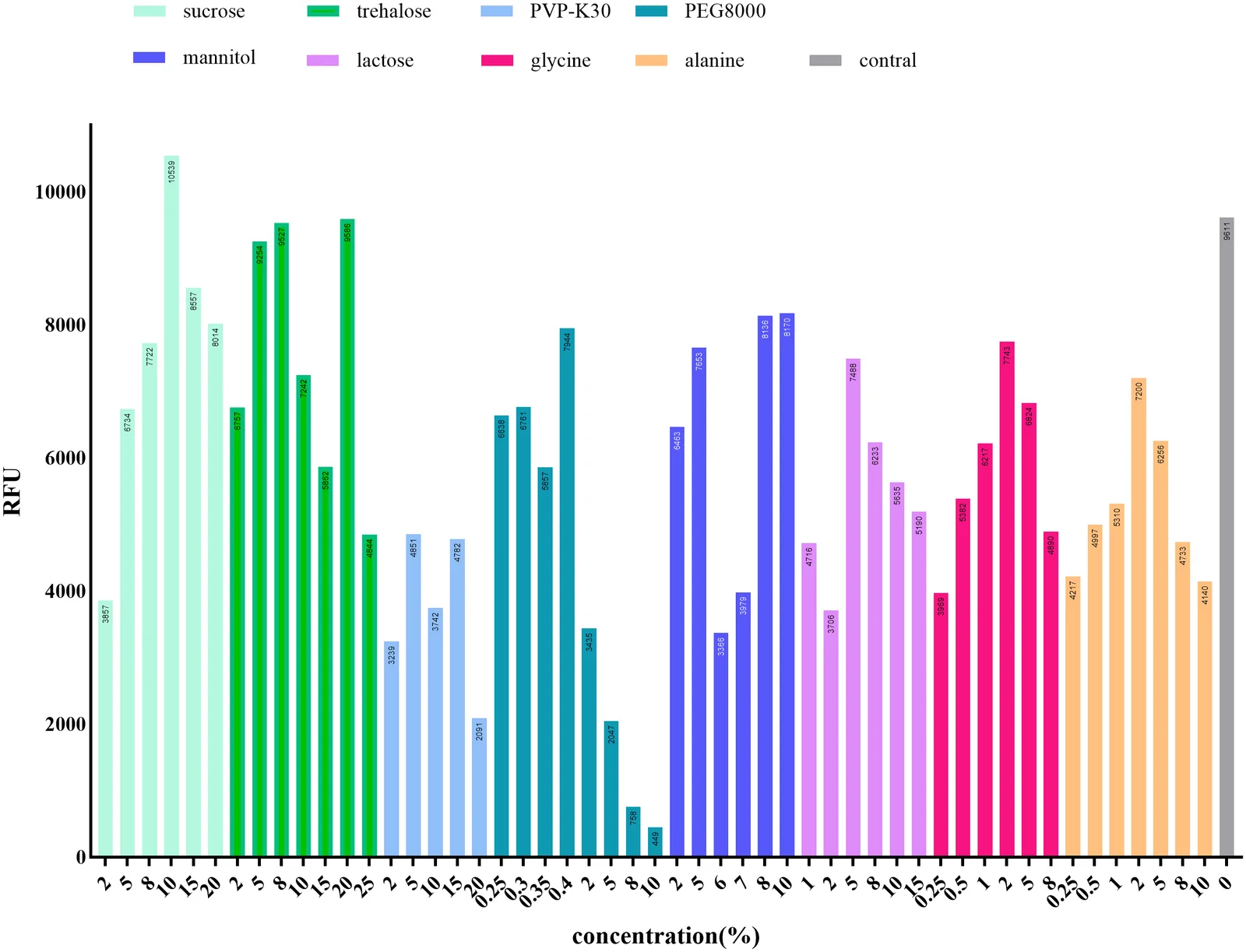
Effects of individual lyoprotectant concentrations on amplification efficiency.

The impact of individual lyoprotectants at varying concentrations on amplification efficiency was assessed by comparing mean peak heights across loci to select the optimal protective formulation. For specific assay concentrations, refer to Table 1 [12–16]. Detailed results are shown in Fig 3. Furthermore, the ratio of amplification buffer to lyoprotectant in the premix directly affects both the lyophilization outcome and the final amplification efficiency of the reagent. While a high concentration of amplification buffer may improve amplification performance, it can adversely affect the post-lyophilization morphology of the reagent. Conversely, if the concentration of the lyoprotectant is excessive, although it may improve lyophilization results, it can reduce peak efficiency in subsequent amplification or even render the reagent entirely unsuitable for amplification. Accordingly, this study tested the concentration ratio between the two components. In a 10 μL system, ratios of 1:9, 2:8, 3:7, 4:6, 5:5, 6:4, 7:3, 8:2 and 9:1 were evaluated, and the test results are shown in S7 Fig. Based on the experimental results, the optimal ratio of 4:6 was selected for subsequent single-protectant concentration testing.

Based on the results, among all lyoprotectants tested, only 10% sucrose was found to increase the mean peak height of the loci. Trehalose at 8% and 20% had almost no effect on peak height, whereas all other protectants showed varying degrees of inhibition. After repeated testing, optimization, and confirmation that lyophilized reagent could form a satisfactory structure, a lyoprotectant system with minimal impact on amplification efficiency was selected. The final formulation chosen for the 10 μL system consisted of sucrose [6% (w/v)], trehalose [5% (w/v)], mannitol [4.8% (w/v)], and PEG8000 [3% (w/v)]. Quantities were scaled up proportionally for larger volumes.

**Fig 4:**
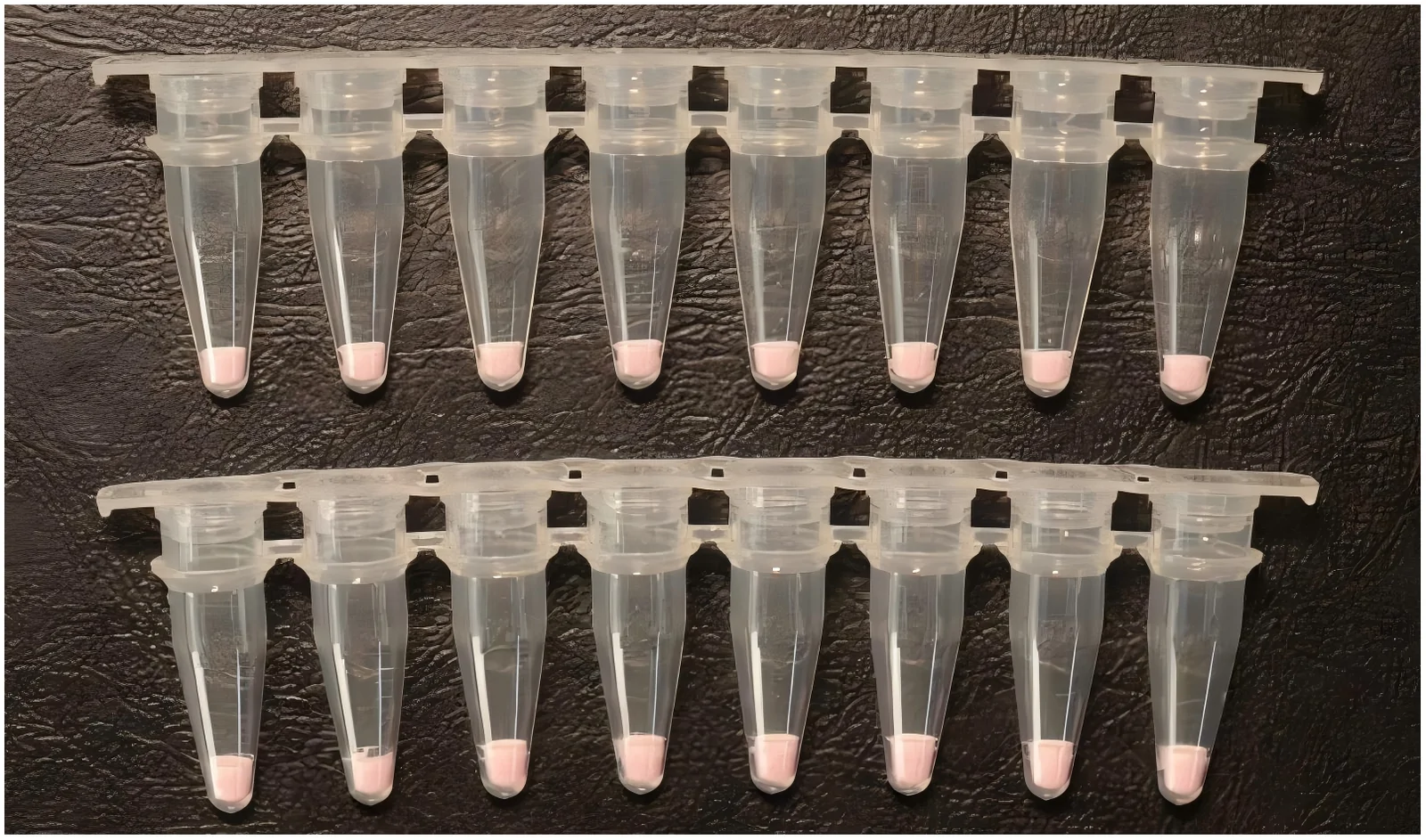
Photographs of in situ lyophilized reagents with volumes of 10 μL and 15 μL. The top row shows the physical image of 10 μL in situ lyophilization, and the bottom row shows 15 μL.

After lyophilization, the samples were retrieved, vacuum-sealed under conditions of <20% ambient humidity, and stored in aluminum foil bags. Furthermore, to meet practical forensic casework requirements, in-situ amplification volumes of 10 μL, 15 μL, and 20 μL were established. Fig 4 shows some of the physical photographs. However, as the lyophilized beads in these formats tended to dislodge from the tubes upon opening due to electrostatic adhesion, the decision was made to retain the in situ lyophilized format for all volumes.

### 3.3 Sensitivity

**Fig 5:**
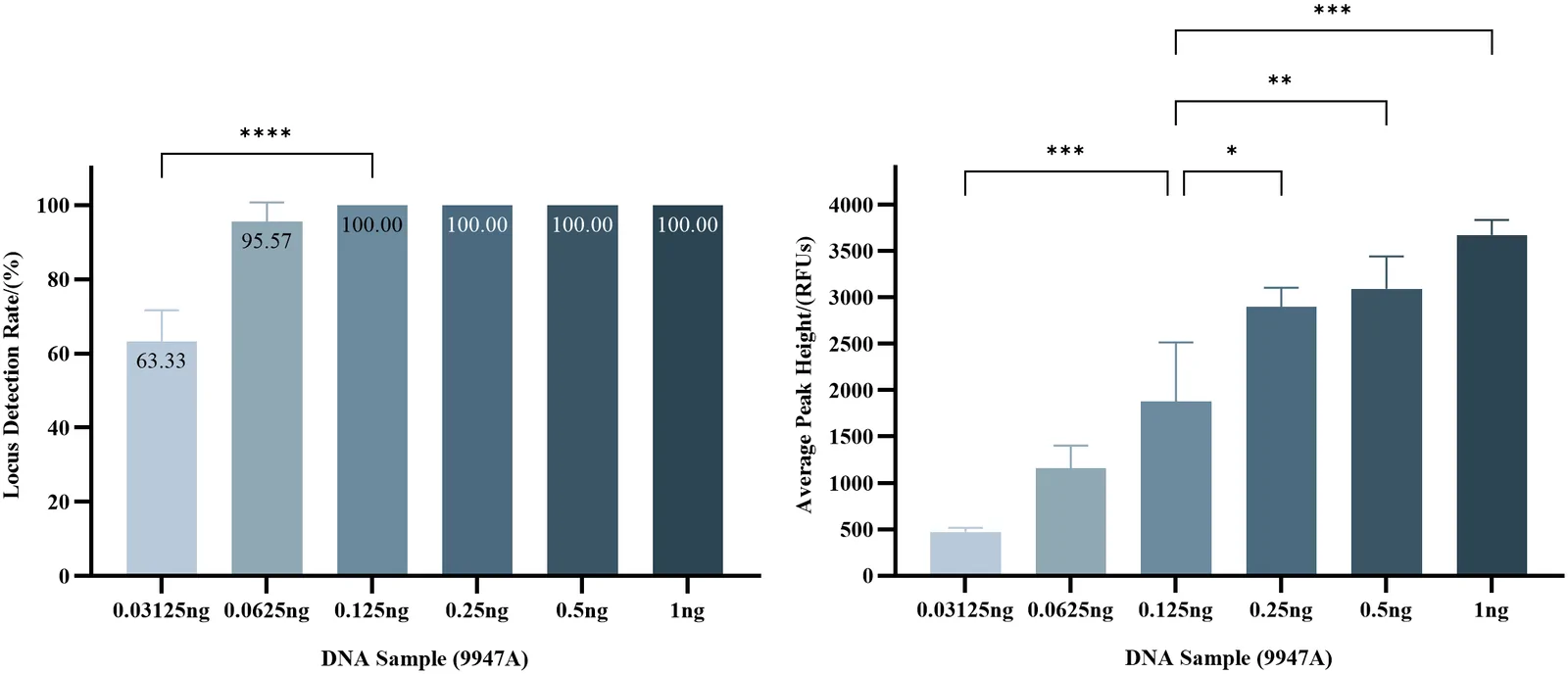
Sensitivity testing results. Mean peak heights of 30 loci at various DNA inputs (n = 3 per concentration). Data are presented as mean ± SD. Comparisons were performed using one-way ANOVA with Dunnett's post hoc test against the 0.125 ng group as the reference. ns, not significant,*p < 0.05,**p < 0.01,***p < 0.001,****p < 0.0001.

The sensitivity of the lyophilized STR reagent was assessed using serial dilutions of 9947A DNA (1, 0.5, 0.25, 0.125, 0.0625, and 0.03125 ng). Statistical analysis was performed using one-way ANOVA with Dunnett's post hoc test, with the 0.125 ng group as the reference. Normality was confirmed by the Shapiro-Wilk test (p > 0.05), and homogeneity of variance was verified by Brown-Forsythe's test (F(5,12) = 0.5368, p = 0.7451). Compared to the 0.125 ng reference group (mean peak height = 1878 RFU), the 0.03125 ng group showed a significant decrease (mean = 471.5 RFU; mean difference = 1407 RFU, 95% CI [622.2, 2191], adjusted p < 0.001). The 0.0625 ng group (1155 RFU) did not differ significantly (mean difference = 723.2 RFU, 95% CI [−61.40, 1508], adjusted p = 0.0742), but one locus (D22S1045) failed to amplify. Higher DNA inputs (0.25–1 ng) showed no significant reduction compared with the 0.125 ng group (adjusted p > 0.05 for all). Based on these results, the sensitivity of the lyophilized reagent was determined to be 0.125 ng. Detailed results are shown in Fig 5.

### 3.4 Stability

**Fig 6:**
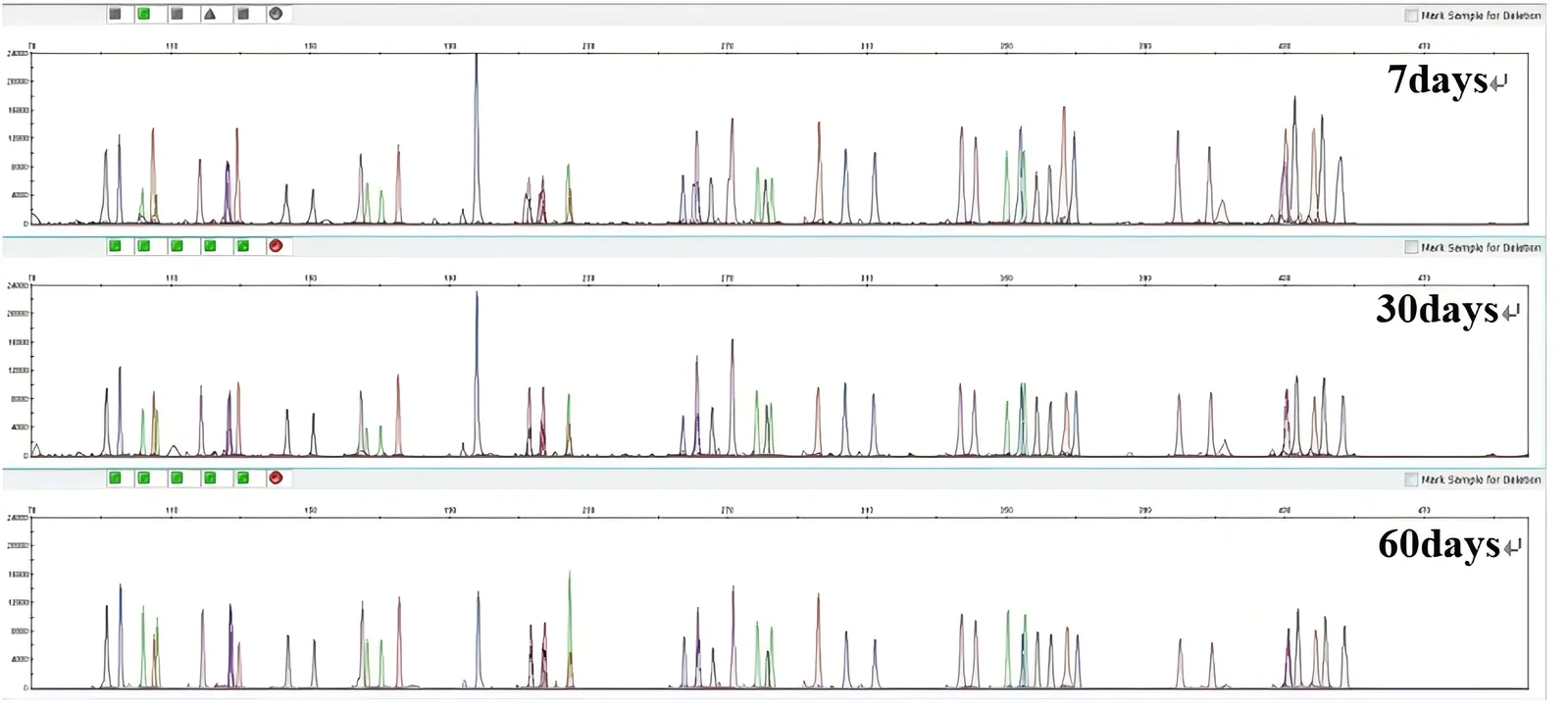
Room-temperature stability test. Locus detection rates and mean peak heights of lyophilized reagents stored at room temperature for 7, 30, and 60 days, compared with liquid reagents stored at −20°C.

Lyophilized reagents from the same batch were stored at room temperature for 7, 30, and 60 days. Using 1 ng of 9947A standard DNA as the template and liquid reagents stored at −20°C as positive controls, the locus detection rates and peak heights were evaluated. After 60 days of room-temperature storage, the lyophilized reagent maintained a 100% locus detection rate, comparable to the liquid reagent stored at −20°C. Visual inspection of the electropherograms showed similar amplification patterns between the two conditions, suggesting that the lyophilized reagent retains adequate amplification efficiency during short-term ambient storage. Formal statistical equivalence testing will be performed in future long-term stability studies. Long-term stability studies remain ongoing (Fig 6).

### 3.5 Species specificity

The results indicate that the system demonstrated acceptable specificity. Among the 13 non-human samples tested, a peak was observed only at the gender-identification locus (amelogenin) for the monkey sample; no peaks were detected at the other 29 loci. The remaining 12 non-human samples all yielded negative results, confirming the system's consistent species specificity (S8 Fig).

### 3.6 Mixture samples

This study conducted mixture tests using female 9947A and male 9948 samples blended in the following ratios: 19:1, 18:2, 16:4, 14:6, 12:8, 10:10, 8:12, 6:14, 2:18, and 1:19. The detection rate for the minor contributor was calculated as the proportion of successfully typed loci out of 30 loci across three replicates (total 90 observations per ratio), and 95% CI were computed using the Clopper-Pearson exact binomial method. Detectable profiles for both major and minor components were obtained at 4:1 ratios, whereas the minor component became difficult to detect when the ratio reached 9:1. For detailed results, refer to Table 6.

**Table 6:** Mixed sample test results.

Mixture ratio	The DNA template composition		Detection rate for the minor contributor	95% CI
	9947A (ng/μL）	9948 (ng/μL）		
**19:1**	0.95	0.05	3.33%	0.7-9.4
**18:2**	0.9	0.1	20%	12.3-29.8
**16:4**	0.8	0.2	100%	96.0-100.0
**14:6**	0.7	0.3	100%	96.0-100.0
**12:8**	0.6	0.4	100%	96.0-100.0
**10:10**	0.5	0.5	100%	96.0-100.0
**8:12**	0.4	0.6	100%	96.0-100.0
**6:14**	0.3	0.7	100%	96.0-100.0
**4:16**	0.2	0.8	100%	96.0-100.0
**2:18**	0.1	0.9	33.3%	23.7-44.1
**1:19**	0.05	0.95	0%	NE^a^

^a^NE:Not estimable. The 95% CI could not be computed because no events were detected across all replicates.

### 3.7 Inhibition

The study examined the system’s tolerance to inhibitors (hemin, indigo, humic acid, and EDTA). Based on the experimental results, the maximum tolerance levels of the system were determined to be 0.25 mmol/L for hematin and EDTA, 3 mmol/L for indigo, and 60 ng/μL for humic acid (S9 Fig).

### 3.8 Simulated trace sample analysis capability

**Fig 7:**
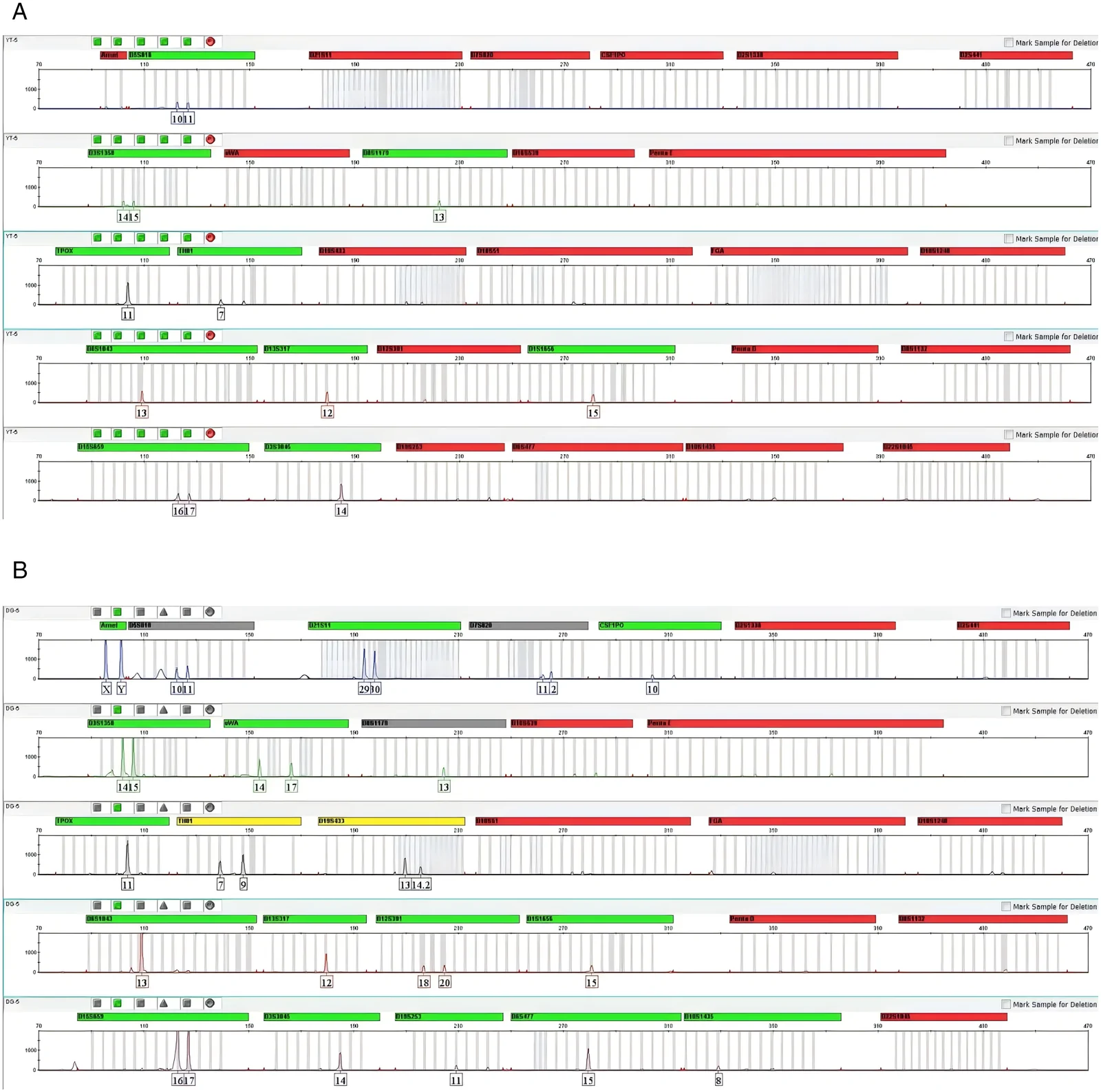
Comparison of trace sample detection performance. A. Liquid reagent; B. Lyophilized reagent.

DNA extracted from bloodstain cards from actual casework using the QIAGEN MagAttract® M48 DNA Manual Kit (QIAGEN, Hilden, Germany) served as the template, and quantification was performed using Qubit (Thermo Fisher Scientific, Woodlands, Singapore). The measured sample concentration was about 0.026 ng/μL. Amplification was conducted using both 10 μL lyophilized and liquid reagents. The lyophilized reagent employed full-volume loading (i.e., 10 μL of sample), whereas the liquid reagent, due to volume constraints imposed by the 5 × buffer and 2.5 × primer, accommodated a maximum of 4 μL of sample. The results demonstrated that, compared to the maximum loading volume of the liquid reagent, full-volume loading with the lyophilized reagent enhanced both the locus detection rate and the average peak height across loci (Fig 7). The mean peak height was 1456.7 ± 142.9 (n = 3) for the lyophilized reagent and 1001.0 ± 82.53 (n = 3) for the liquid reagent. The mean difference between the two reagents was 455.7 (95% CI [100.7, 810.8]). Paired t-test yielded t (2) = 5.522, P = 0.0313 (P < 0.05), indicating that the lyophilized reagent produced significantly higher detection signal intensity than the liquid reagent.

### 3.9 Parallel comparison using liquid reagents

**Fig 8:**
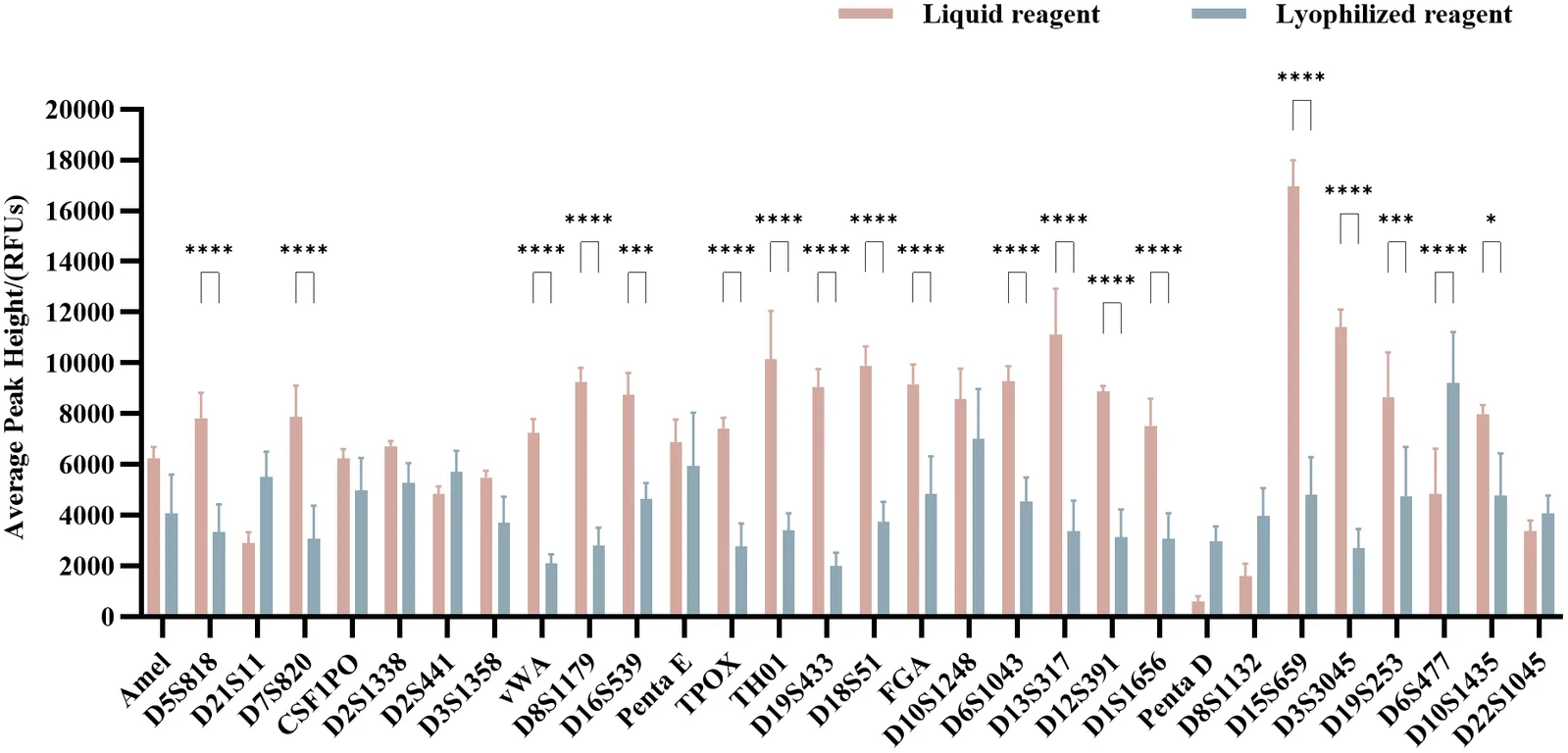
Amplification efficiency of loci was compared between lyophilized and liquid reagents. *P < 0.05, ***P < 0.001, ***P < 0.0001.

Amplification systems (10 μL) for the two reagent types were prepared according to the specific component volumes listed in Table 2. Experimental results indicated that, compared with liquid reagents, the overall amplification efficiency of the lyophilized reagent decreased after lyophilization, due to unavoidable process-related effects on the DNA polymerase. However, both reagents achieved a 100% locus detection rate, meeting the requirements for forensic analysis (Fig 8). Detailed statistical results for all 30 loci are provided in Supplementary S1 Table.

## 4. Discussion

This study successfully developed and validated a lyophilized PCR kit for 30 autosomal STR loci. It achieved 100% detection with 0.125 ng DNA and demonstrated good room-temperature stability, offering a cold-chain advantage. The kit showed high specificity, detected minor components in 4:1 mixtures, and tolerated common inhibitors. Its key benefit for trace samples is enabling full-volume loading, boosting sensitivity versus volume-restricted liquid reagents. While slightly less efficient than liquid reagents at some loci, it maintained 100% detection, meeting all forensic requirements.

During the development of the lyophilized system, we focused on optimizing components,including the premix, enzymes, and primers. By removing glycerol, screening DNA polymerases suitable for lyophilization, and redesigning primers for specific loci, the amplification efficiency of the lyophilized reagents was successfully improved.

In selecting the lyophilization process, we compared two technical approaches: in situ lyophilization and bead-based lyophilization. In situ lyophilization involves directly adding the entire mixture by volume into 8-well strips for freeze-drying, whereas bead-based lyophilization requires pre-freezing the mixture droplets in liquid nitrogen before lyophilization. Although under the same lyophilization conditions, bead-based lyophilization theoretically yields better morphology due to the lower pre-freezing temperature achieved with liquid nitrogen, it faces practical challenges, such as electrostatic adhesion that can cause beads to detach from the strips, difficulty in forming small-volume beads, and the need for secondary transfer after lyophilization. Therefore, considering operational simplicity, stability, and better suitability for rapid on-site application, this study ultimately favored the in-situ lyophilization system.

In screening protective agent systems, testing individual protective agents provided general guidance. The key optimization strategy involved modifications to the amplification system. Notably, since glycerol has been documented to affect freeze-dried morphology, the selected DNA polymerase and buffer in this system were ensured to be glycerol-free [7,17].

Although the freeze-drying process inevitably impacts enzyme activity, this effect can be effectively mitigated by selecting lyophilization-compatible enzymes and employing a composite freeze-drying protective agent to shape, fill, and adjust pH. Building on this, through optimization and proportional adjustment of the amplification buffer and protective agent system, a final formulation—consisting of 6% sucrose, 5% trehalose, 4.8% mannitol, and 3% PEG 8000 in a 4:6 ratio with the buffer—was determined. This formulation enables the formation of a stable freeze-dried matrix and delivers favorable amplification efficiency.

In summary, the advantages of lyophilized reagents are as follows: Firstly, they enable higher template loading, making them suitable for trace samples. After the buffer and primers are lyophilized, the lyophilized reagents enable whole-volume loading of the template. Furthermore, the stability of the short-term lyophilized reagents at ambient temperature was preliminarily confirmed, which facilitates their transportation and storage. The low moisture content in the dried state helps maintain system stability, enabling long-term storage and providing a feasible solution for resource-limited settings. Additionally, the operation is simple and reduces contamination risks. Lyophilized reagents are reconstituted with water before adding the DNA template, effectively minimizing contamination caused by improper handling or environmental factors.

For the lyophilized STR reagent developed in this study, future work could be further extended in the following directions to enhance its performance and broaden its application scope. First, the protective agent formulation and lyophilization process could be further optimized—for instance, by testing novel protective agents or adjusting lyophilization program parameters to minimize loss of key components, such as enzyme activity, during freeze-drying, thereby further improving the reagent’s amplification efficiency and stability. Second, more systematic and diverse sample validation should be conducted, including testing of highly degraded samples, complex mixtures, and actual case materials contaminated with common inhibitors encountered at crime scenes, to comprehensively evaluate its reliability and robustness in complex real‑world environments. Third, efforts should be made to integrate the reagent with rapid detection technology platforms, such as combining it with field‑deployable devices,such as microfluidic chips, to build an integrated “sample‑in‑answer‑out” rapid detection system, truly enabling instant DNA identification at crime scenes or in emergency scenarios [17,18]. Finally, the lyophilization process system established in this study could be extended to other important forensic genetic marker systems, such as single‑nucleotide polymorphisms, Y‑chromosome STRs, or microhaplotypes, thereby forming a series of ambient‑stable forensic genetic freeze‑dried reagents that provide flexible and convenient technical solutions for multidimensional identity recognition and complex kinship analysis.

## 5. Conclusion

This study successfully developed and validated a lyophilized PCR kit for 6-dye multiplex amplification of 30 autosomal STR loci. Through systematic optimization of the liquid reagent system (buffer, enzyme, primers), screening of composite lyoprotectants, and refinement of the lyophilization process, the developed reagent enables room-temperature storage and transportation, simplified operation (ready-to-use upon rehydration), high sensitivity (0.125 ng, supporting full-volume loading to enhance detection of trace samples), and short-term stability (about 60 days at room temperature). In summary, the lyophilized reagents successfully overcome the limitations of liquid reagents, advancing forensic DNA testing in terms of accessibility and practicality.

## Supporting information

S1 Fig Determination of Tg’ and Teu for the lyoprotectant system. (PDF)

S2 Fig Map of DNATyper^TM^ 30 liquid/lyophilized reagent loci distribution. (PDF)

S3 Fig Activity of six Taq DNA polymerases before and after lyophilization. (PDF)

S4 Fig Enzyme dosage optimization. (PDF)

S5 Fig Annealing temperature optimization. (PDF)

S6 Fig Optimization of the thermal cycling parameters. (PDF)

S7 Fig Optimization of the formulation ratio between amplification buffer and lyoprotectant. (PDF)

S8 Fig Specificity test results (13 non-human samples). (PDF)

S9 Fig Inhibitor tolerance test results. (PDF)

S1 Table Peak height comparison between lyophilized and liquid reagents for each of the 30 STR loci. (PDF)
