# Supplementary material for: Development and validation of the 6-dye lyophilized fluorescent multiplex PCR system
Source: PLoS One. 2026 Aug 27;21(8):e0356258. doi: 10.1371/journal.pone.0356258 (PMC13521347; doi:10.1371/journal.pone.0356258)
Supplement: Supplementary file 1 [file pone.0356258.s001.pdf]

S1 Fig. Determination of Tg' and Teu for the lyoprotectant system.

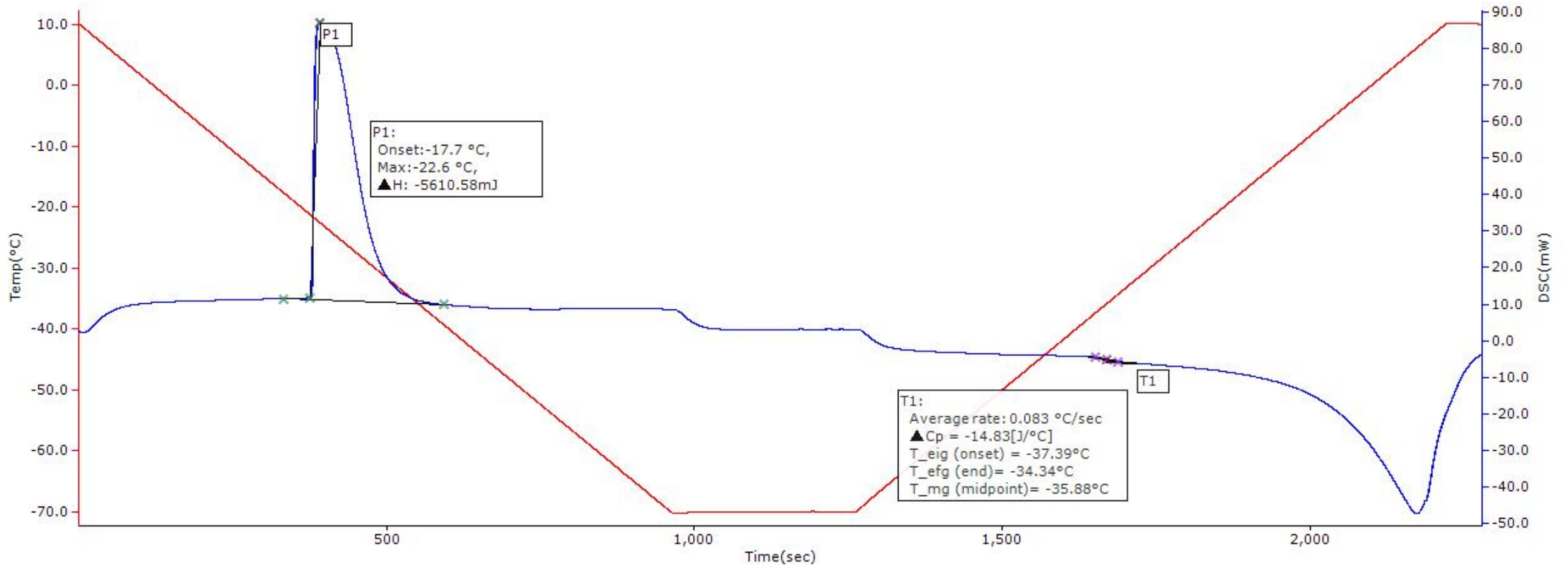

Teu : -22.6℃ Tg' : -35.88℃

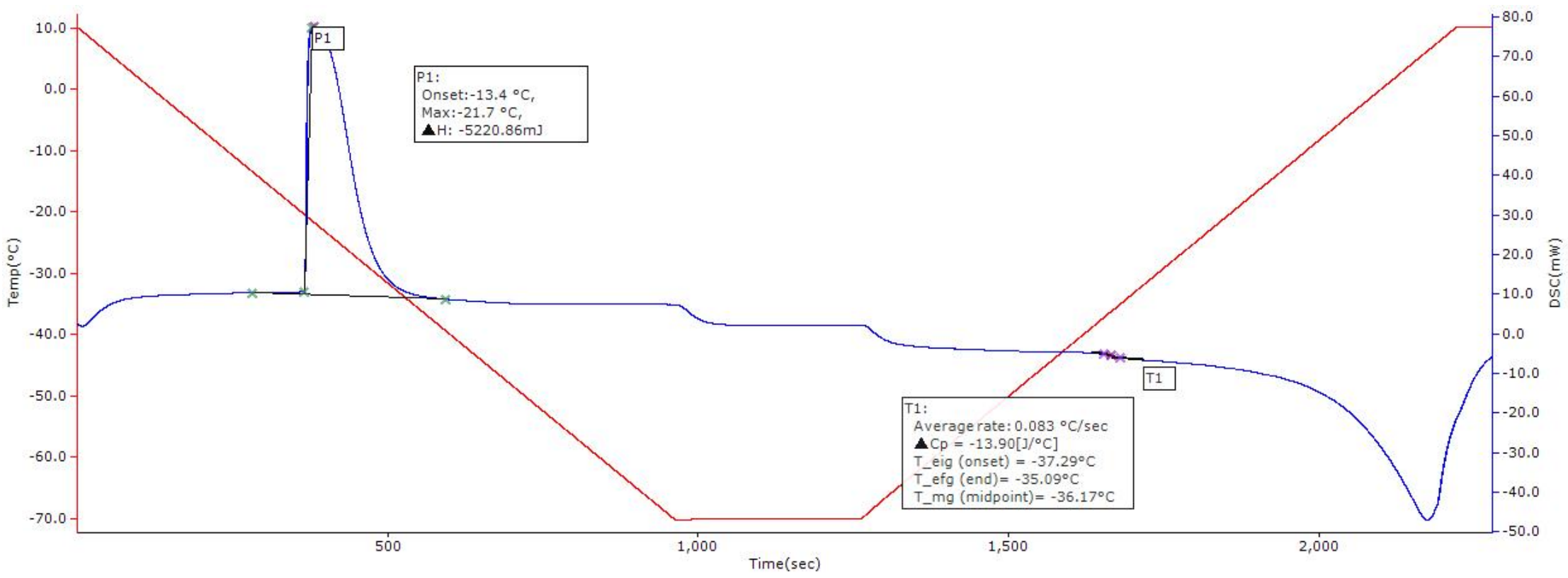

Teu : -21.7℃ Tg' : -36.17℃

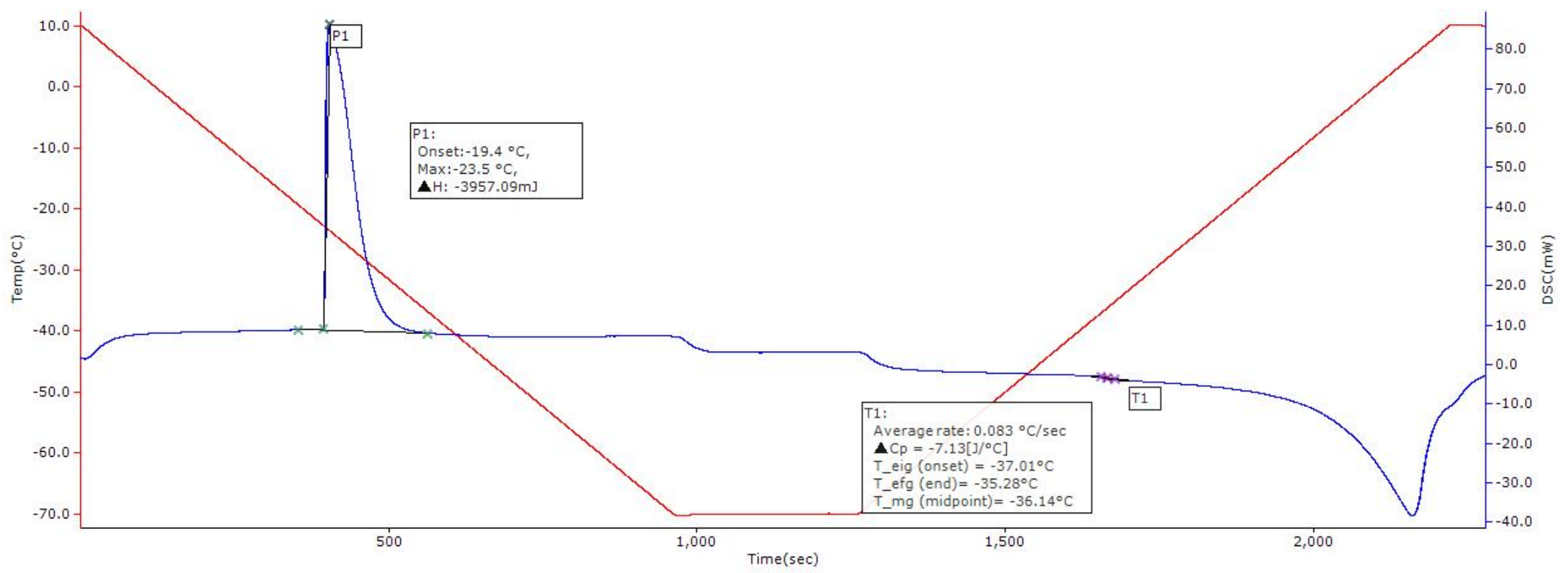

Teu : -23.5℃ Tg' : -36.14℃
