# Supplementary material for: Development and validation of the 6-dye lyophilized fluorescent multiplex PCR system
Source: PLoS One. 2026 Aug 27;21(8):e0356258. doi: 10.1371/journal.pone.0356258 (PMC13521347; doi:10.1371/journal.pone.0356258)
Supplement: Supplementary file 3 [file pone.0356258.s003.pdf]

S3 Fig. Activity of six Taq DNA polymerases before and after lyophilization.

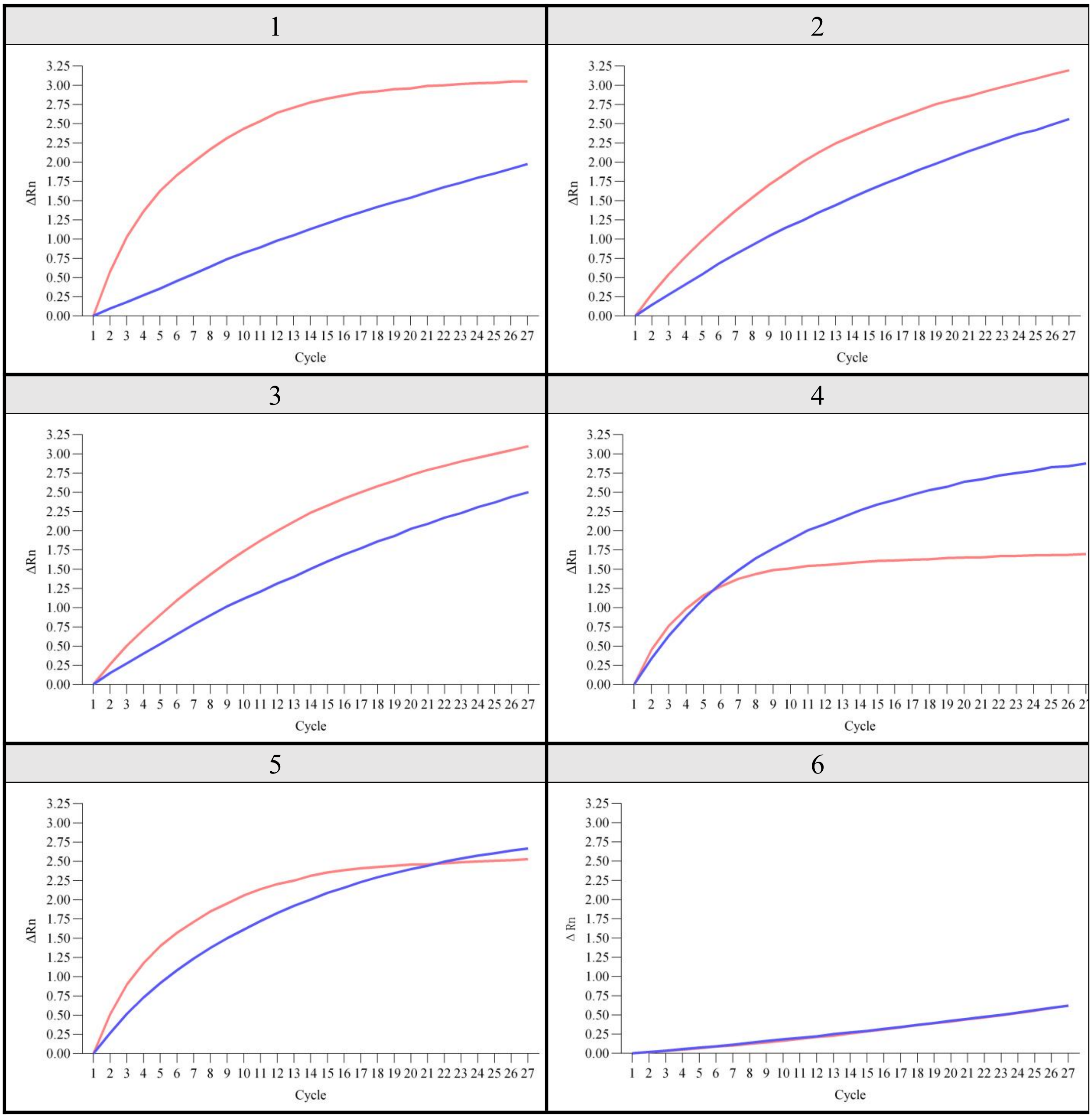

Notes： The fluorescence dye method was used to test the change of enzyme activity before and after freeze-drying. The red line was the enzyme activity before freeze-drying, and the blue line was the enzyme activity after freeze-drying.
