# Supplementary material for: Development and validation of the 6-dye lyophilized fluorescent multiplex PCR system
Source: PLoS One. 2026 Aug 27;21(8):e0356258. doi: 10.1371/journal.pone.0356258 (PMC13521347; doi:10.1371/journal.pone.0356258)
Supplement: Supplementary file 4 [file pone.0356258.s004.pdf]

S4 Fig. Enzyme dosage optimization.

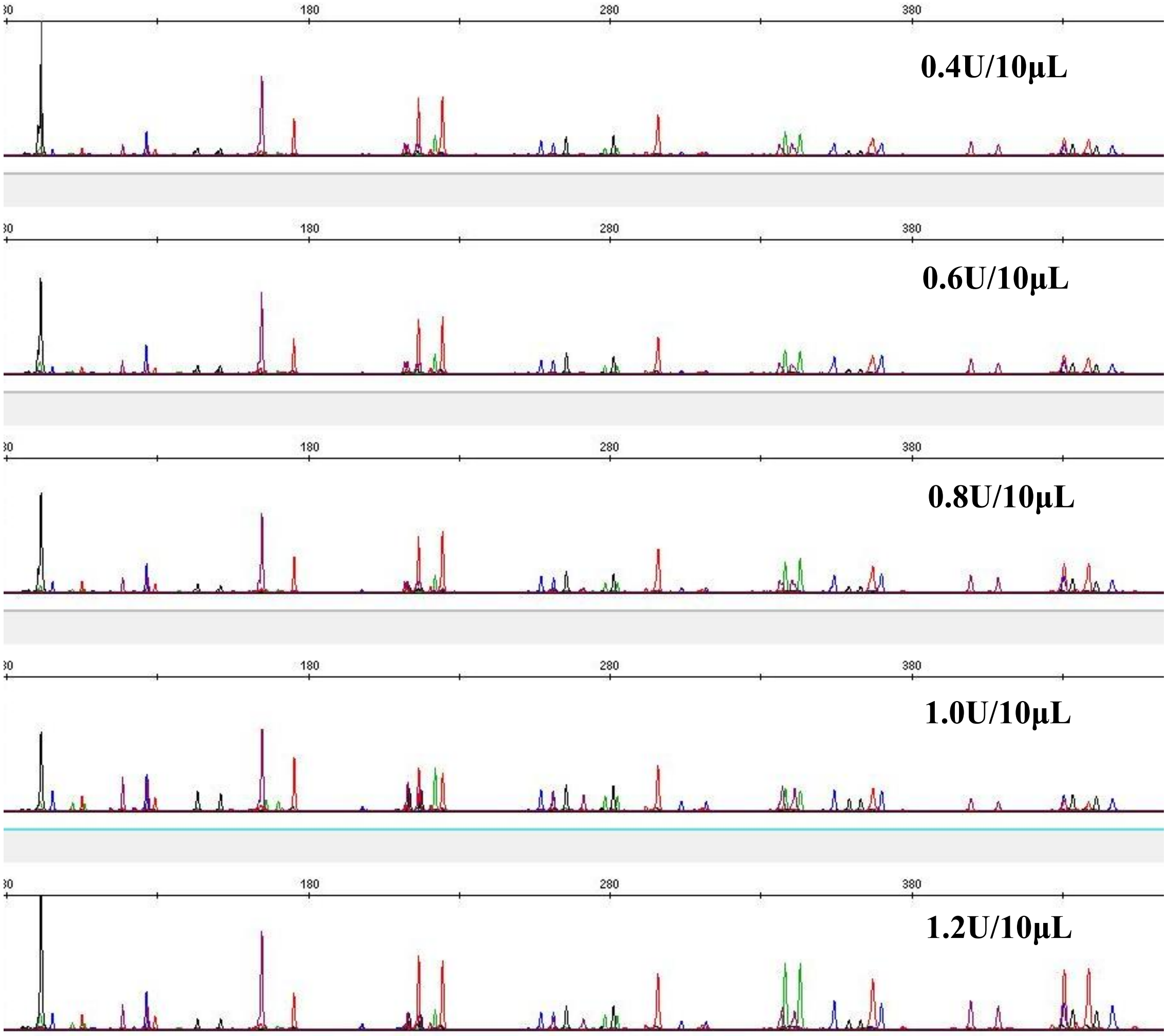

Notes: The enzyme amounts added in the 10μL system range from 0.4U to 1.2U, listed sequentially from top to bottom.
