# Supplementary material for: Development and validation of the 6-dye lyophilized fluorescent multiplex PCR system
Source: PLoS One. 2026 Aug 27;21(8):e0356258. doi: 10.1371/journal.pone.0356258 (PMC13521347; doi:10.1371/journal.pone.0356258)

S6 Fig. Optimization of the thermal cycling parameters.

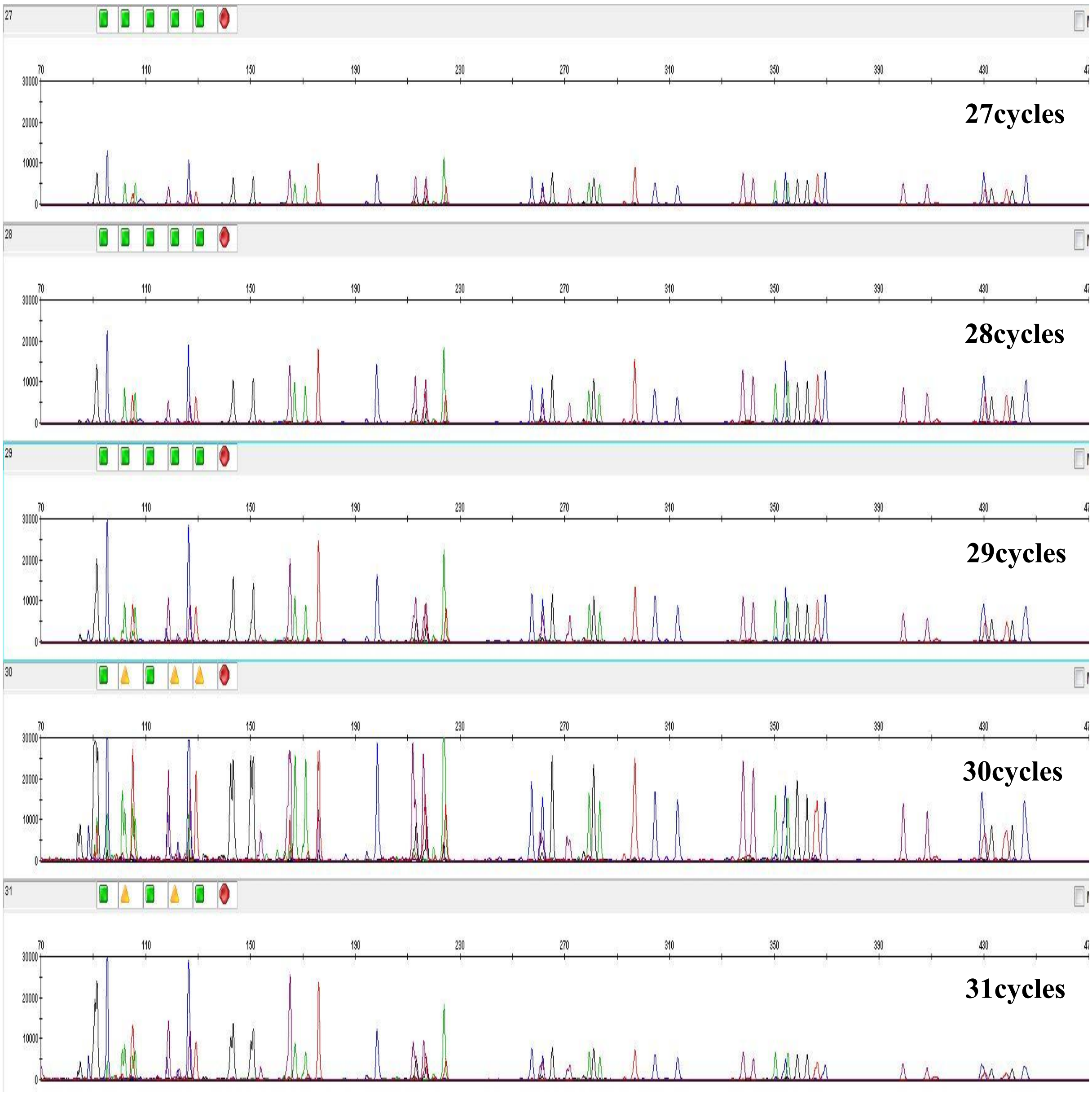

Supplement: Supplementary file 6 [file pone.0356258.s006.pdf]
