# Supplementary material for: Development and validation of the 6-dye lyophilized fluorescent multiplex PCR system
Source: PLoS One. 2026 Aug 27;21(8):e0356258. doi: 10.1371/journal.pone.0356258 (PMC13521347; doi:10.1371/journal.pone.0356258)
Supplement: Supplementary file 7 [file pone.0356258.s007.pdf]

S7 Fig. Optimization of the formulation ratio between amplification buffer and lyoprotectant.

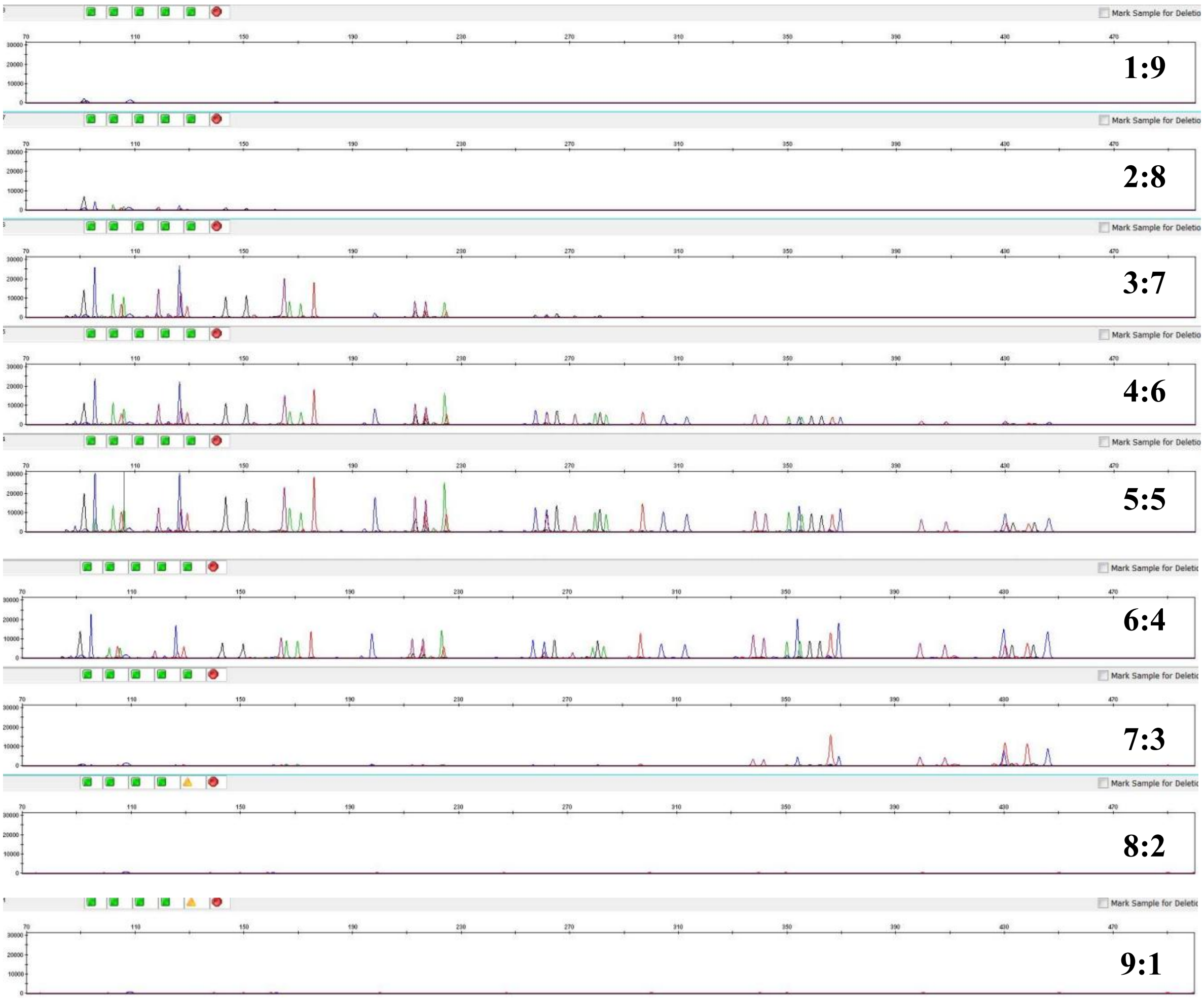

Notes: An excessively high buffer concentration compromised cake morphology, while an excessive lyoprotectant concentration reduced amplification efficiency. A ratio of 4:6 (buffer:lyoprotectant) was selected as the optimal balance.
