# Supplementary material for: Development and validation of the 6-dye lyophilized fluorescent multiplex PCR system
Source: PLoS One. 2026 Aug 27;21(8):e0356258. doi: 10.1371/journal.pone.0356258 (PMC13521347; doi:10.1371/journal.pone.0356258)
Supplement: Supplementary file 8 [file pone.0356258.s008.pdf]

S8 Fig. Specificity test results (13 non-human samples).

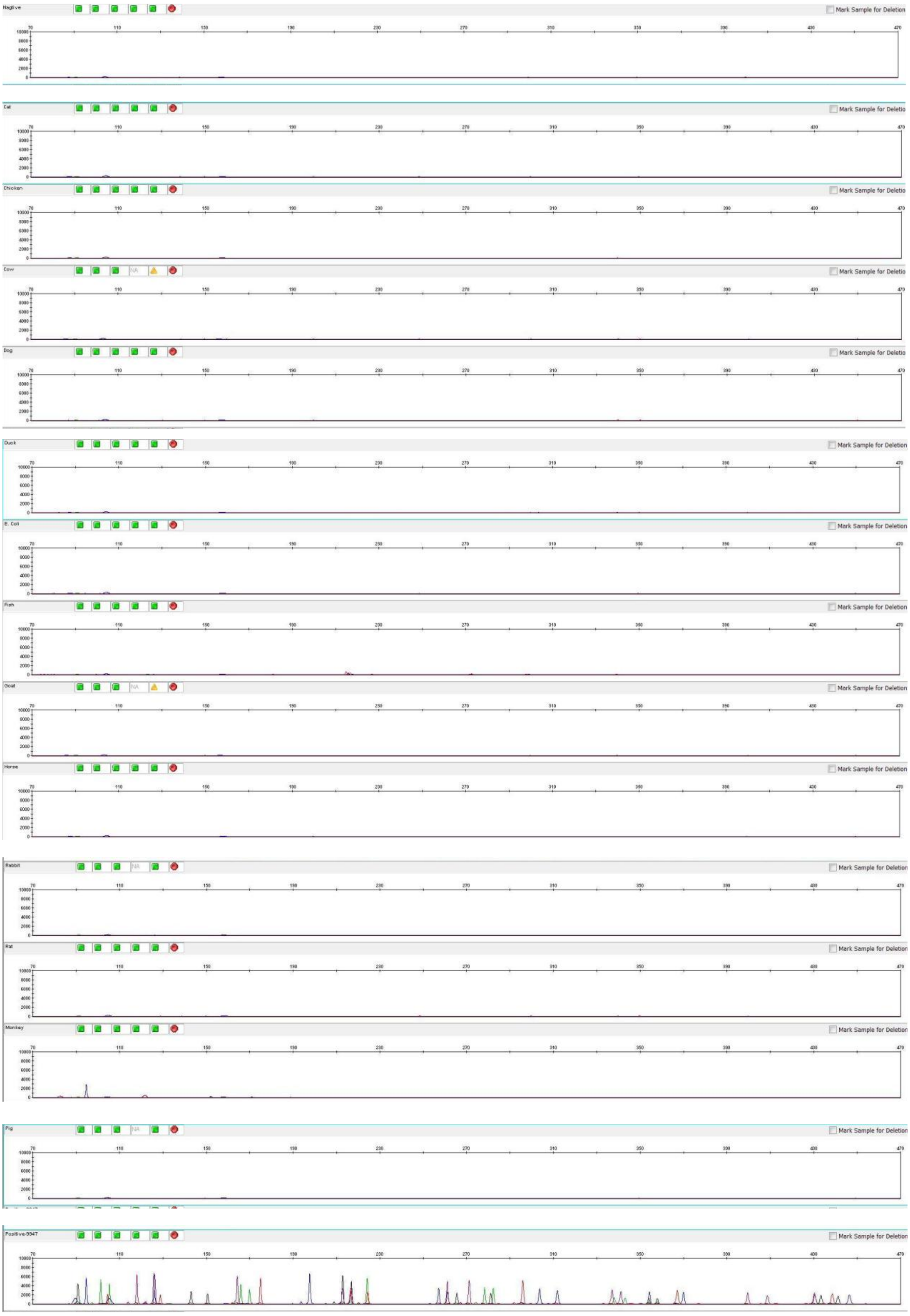

Notes:The samples are arranged from top to bottom as follows: negative control, cat, chicken, cow, dog, duck, *E. coli*, fish, goat, horse, rabbit, rat,

monkey, pig, positive control.
