# Supplementary material for: Development and validation of the 6-dye lyophilized fluorescent multiplex PCR system
Source: PLoS One. 2026 Aug 27;21(8):e0356258. doi: 10.1371/journal.pone.0356258 (PMC13521347; doi:10.1371/journal.pone.0356258)
Supplement: Supplementary file 9 [file pone.0356258.s009.pdf]

S9 Fig. Inhibitor tolerance test results.

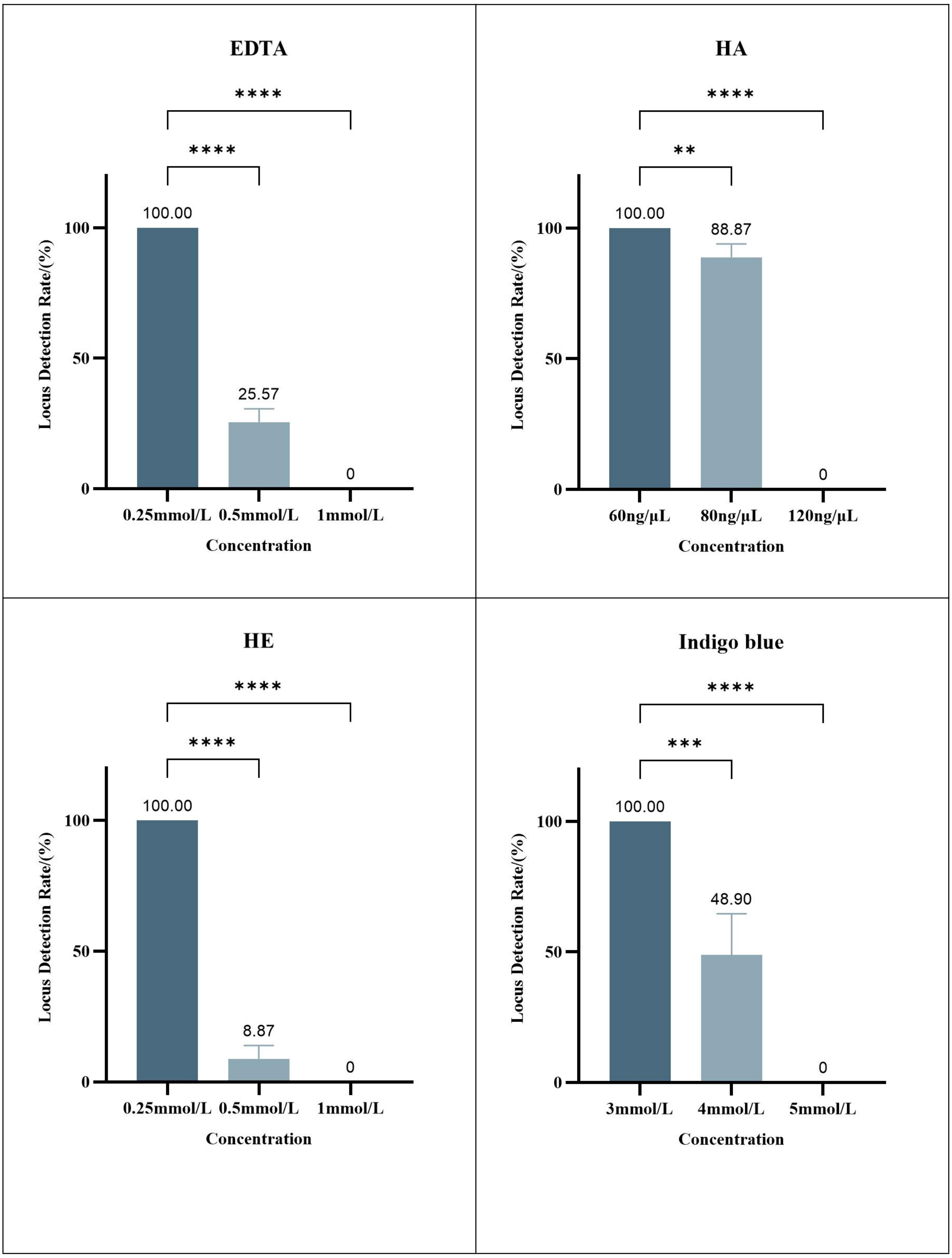

Notes : Data are mean detection rate (%) ± SD from 3 replicates (n=3).** $P < 0.01$,***$P < 0.001$,****$P < 0.0001$.
