# Supplementary material for: Development and validation of the 6-dye lyophilized fluorescent multiplex PCR system
Source: PLoS One. 2026 Aug 27;21(8):e0356258. doi: 10.1371/journal.pone.0356258 (PMC13521347; doi:10.1371/journal.pone.0356258)
Supplement: Supplementary file 10 [file pone.0356258.s010.pdf]

Table S1. Peak height comparison between lyophilized and liquid reagents for each of the 30 STR loci.

Locus	Mean peak height (lyophilized)	Mean peak height (liquid)	Mean difference	95% CI	P Value
**Amel**	6260	4065	2195	[-627.2, 5016]	0.3434
**D5S818**	7824	3338	4486	[1665, 7308]	<0.0001
**D21S11**	2897	5528	-2631	[-5453, 190.8]	0.0962
**D7S820**	7880	3082	4798	[1976, 7619]	<0.0001
**CSF1PO**	6237	4970	1268	[-1554, 4089]	0.9929
**D2S1338**	6732	5277	1455	[-1366, 4277]	0.9583
**D2S441**	4849	5706	-856.7	[-3678, 1965]	>0.9999
**D3S1358**	5486	3707	1780	[-1042, 4601]	0.7503
**vWA**	7247	2111	5136	[2314, 7958]	<0.0001
**D8S1179**	9261	2792	6469	[3647, 9291]	<0.0001
**D16S539**	8737	4638	4099	[1277, 6920]	0.0002
**Penta E**	6870	5958	912.0	[-1910, 3734]	>0.9999
**TPOX**	7422	2757	4665	[1843, 7486]	<0.0001
**TH01**	10146	3397	6749	[3928, 9571]	<0.0001
**D19S433**	9043	1984	7059	[4237, 9880]	<0.0001
**D18S51**	9890	3723	6167	[3345, 8989]	<0.0001
**FGA**	9151	4826	4326	[1504, 7147]	<0.0001
**D10S1248**	8571	7024	1547	[-1275, 4369]	0.9209
**D6S1043**	9273	4523	4750	[1929, 7572]	<0.0001
**D13S317**	11123	3375	7748	[4926, 10570]	<0.0001
**D12S391**	8873	3135	5738	[2917, 8560]]	<0.0001
**D1S1656**	7530	3066	4464	[1642, 7286]	<0.0001
**Penta D**	613.0	2960	-2347	[-5169, 474.8]	0.2296
**D8S1132**	1614	3969	-2355	[-5176, 467.2]	0.2247
**D15S659**	16957	4804	12153	[9331, 14975]	<0.0001
**D3S3045**	11425	2700	8726	[5904, 11547]	<0.0001
**D19S253**	8667	4734	3932	[1111, 6754]	0.0005
**D6S477**	4824	9226	-4402	[-7223, -1580]	<0.0001
**D10S1435**	7985	4783	3202	[380.2, 6024]	0.0120
**D22S1045**	3375	4073	-698.3	[-3520, 2123]	>0.9999
